# Case of Injury to the Head

**Published:** 1874-11

**Authors:** George J. Monroe

**Affiliations:** Leland, LaSalle Co., Ill.


ARTICLE III.—*A Case of Injury of the Head.* By GEORGE J. MONROE, M.D., Leland, LaSalle Co., Ill.

Mr. Iver Jacobson was loading the horse power of a threshing machine on the evening of October 1, 1872, by rolling it up with a roller through which a crow bar was passed. By some means the bar slipped from his hands, when it flew back, having the whole weight of the horse power upon it. The bar struck him upon the top of the head, at once knocking him senseless. I was sent for, and found him, in about one hour after the accident, in a comatose condition, with heavy labored breathing. Pulse, 30; respiration, 7; feet and legs cold; eyes and face swollen. Found a flesh wound on the crown of the head about five inches long, running in the antero posterior direction. Found a fracture extending from the occipital bone to the frontal, and down the frontal to the orbit. About midway from the occipital to the frontal, the parietal bone was fractured on the left side down to the temporal bone, and several pieces loosened. The frontal was also fractured on the left side from the middle fracture, commencing at its contact with the parietal, to the external superior angle of the eye. I shaved the head near the injury, and removed several large pieces of detached bone. Brain matter, I should say, to the extent of two ounces exsuded. Ordered ice to the scalp, jugs of warm water to extremities, as well as about the body. Told his friends that he would die, but if alive in the morning, to notify me; also, to send for Dr. Vosburgh, of Earl, to meet me in consultation. Was informed in the morning he was still alive, and that Dr. Vosburgh had been sent for. Took Dr. Hinkley from our town, and reached the patient about nine A. M. Dr. Vosburgh soon arrived. Found the following conditions: pulse, 60; respiration, 15; extremities warm; reaction taking place; face very much swollen; had passed fœces involuntarily. Drew the urine with catheter. Brain matter still exsuding. The left anterior quarter of the parietal bone was depressed below the balance of the bone at least one-fourth of an inch. Inserted a spatula under it, and pried over the right parietal bone, which means was successful in elevating the bone to its proper position. Instead of it falling back, as I anticipated it would, on removal of the spatula, it remained as elevated, and never has become again depressed.

Not deeming any further interference necessary, expecting our patient to die, we ordered ice to be continued to the head, and heat to the extremities. As he could not swallow, we ordered injections of beef tea and quinine per rectum.

Oct. 3. Head very hot; pulse, one hundred and ten; respiration, thirty-five. Had not voluntarily moved any portion of the body. Continue treatment.

Oct. 4. Found patient in nearly same condition. His wife said that some liquid she had put in his mouth, after remaining some time, had been swallowed. I tried the experiment, and after retaining liquid which I put in his mouth some three minutes, he swallowed it.

The symptoms remained very much the same for the next seven days, with the exception of the swallowing, which grew better and better each day, until about the twelfth, when he would swallow any liquid which was put in his mouth. On the thirteenth, he opened his eyes, but I do not think he could see. Along about this time he passed his urine without assistance. He moved his hands and feet some; made a great many motions with his hands, as though he wanted something. I concluded he had partial control over the rectum, and his returning sensation told him his bowels wanted to be emptied. His wife put cloths under him, and during my visit he had an evacuation. After this, when he made those peculiar signs, his wife would put cloths under him, and his bowels would move. I think sight partially returned about the sixteenth. On putting a piece of cracker into his mouth at this date, he attempted to chew it. The fever had entirely subsided on the twentieth; the external wound nearly healed. A bunch about the size of a walnut had formed where union had taken place in the scalp. On examination, I concluded I felt fluctuation. I struck my lancet into this swelling, when there was a profuse dicharge of purulent matter. In about an hour from this time, his wife, on going back into the room, from which she had been a few minutes absent, found him sitting up in bed and looking bewildered. He asked, after looking at her awhile, where he was. She told him. He then asked if they had gone with the separator. She told him, yes. He then asked where the horse power was. She told him it was gone too. This was apparently a poser to him, for he said he thought he was helping to load it.

He finally put his hand to his head, and found it sore. He asked how that happened. His wife then told him all about the accident. He could not comprehend that he had been lying there twenty-one days. It was all lost time to him, and something of a mystery. I found him rational on my next visit, but with a difficulty of everything appearing double when looked at with the left eye. He continued to improve from day to day. The difficulty with vision gradually subsided, and was all gone in about six months. He frequently tells me that three weeks of his life was a blank. The wonder is, that with such an extensive fracture of the skull, with such an extent of brain injury and loss of brain matter, he recovered at all; and still more wonderful, that he should recover without any impairment of his intellectual faculties.

